# A global comparative study of wealth-pain gradients: Investigating individual- and country-level associations

**DOI:** 10.1016/j.dialog.2023.100122

**Published:** 2023-03-08

**Authors:** Zachary Zimmer, Anna Zajacova, Kathryn Fraser, Daniel Powers, Hanna Grol-Prokopczyk

**Affiliations:** a Department of Family Studies and Gerontology, Global Aging and Community Initiative, 166 Bedford Highway, McCain Centre 201C, Halifax, Nova Scotia B2M2J6, Canada; b Department of Sociology, Social Science Centre Room 5306, University of Western Ontario, London, Ontario N6A5C2, Canada; c Global Aging and Community Initiative, 166 Bedford Highway, McCain Centre 201C, Halifax, Nova Scotia B2M2J6, Canada; d Department of Sociology, RLP 2.622J, University of Texas at Austin, Austin, TX 78712-1086, USA; e Department of Sociology, 430 Park Hall, University at Buffalo, Buffalo, NY 14260-4140, USA

**Keywords:** Comparative research, Global Health, Health gradient, multilevel modeling, Pain, World Health Study

## Abstract

Pain is a significant yet underappreciated dimension of population health. Its associations with individual- and country-level wealth are not well characterized using global data. We estimate both individual- and country-level wealth inequalities in pain in 51 countries by combining data from the World Health Organization's World Health Survey with country-level contextual data. Our research concentrates on three questions: 1) Are inequalities in pain by individual-level wealth observed in countries worldwide? 2) Does country-level wealth also relate to pain prevalence? 3) Can variations in pain reporting also be explained by country-level contextual factors, such as income inequality? Analytical steps include logistic regressions conducted for separate countries, and multilevel models with random wealth slopes and resultant predicted probabilities using a dataset that pools information across countries. Findings show individual-level wealth negatively predicts pain almost universally, but the association strength differs across countries. Country-level contextual factors do not explain away these associations. Pain is generally less prevalent in wealthier countries, but the exact nature of the association between country-level wealth and pain depends on the moderating influence of country-level income inequality, measured by the Gini index. The lower the income inequality, the more likely it is that poor countries experience the highest and rich countries the lowest prevalence of pain. In contrast, the higher the income inequality, the more nonlinear the association between country-level wealth and pain reporting such that the highest prevalence is seen in highly nonegalitarian middle-income countries. Our findings help to characterize the global distribution of pain and pain inequalities, and to identify national-level factors that shape pain inequalities.

## 1. Introduction

Pain is an often-underappreciated health challenge [1,2]. However, its importance as an indicator of population health is increasingly being recognized [3,4]. Justification of this lies in the ubiquity of pain as a global health problem and growing concerns about the implications of chronic pain for productivity, quality of life, and costs related to health care and productivity [5-11]. This makes pain very clearly a global population health challenge, and renders identifying factors associated with the distribution of pain within populations essential for understanding disparities in global health [5,12].

Medical problems linked to pain reporting are frequently associated with socioeconomic conditions [13-17]. But chronic pain is also considered to be a condition in its own right [18,19]. International comparative research suggests substantial differences in strength of the socioeconomic status-health associations across countries [20-26]. However, little is known about what drives these differences. Cross-national comparisons tend to focus on a small number of countries, often two or three, or on a particular region, like Europe. Comparisons across countries with different social, political and economic structures are non-existent.

Relative level of wealth is connected with living environment, the ability to purchase health-supporting good and services, and is strongly linked to other indicators of socioeconomic status [27,28]. But whether individual wealth allows for access to health-related resources also depends on their availability, which is enhanced in richer countries [29,30]. Studies show a connection between country-level wealth and population health [31-35]. Therefore, *country*- as well as individual-level wealth may influence inequalities in pain prevalence.

While both individual- and country-level wealth may impact on pain prevalence, there are additional macro-level factors that may impact, explain and/or moderate the way in which wealth and pain are associated. There is a large body of literature exploring how health may be explained by country-level contextual characteristics that describe a country's structural conditions. These factors may represent socioeconomic, political, cultural, or environmental circumstances such as poverty rates, pollution, density, education levels, government policies, health expenditures, labor force factors, among others [26,36-42]. There is also an important line of debate around how population health may be impacted upon by the distribution of wealth [31,33,35,43-45]. The current study expands this literature by examining the association between wealth and pain across a broad set of countries, asking several specific questions. Is there a consistent association between individual-level wealth and pain prevalence worldwide? Does country-level wealth also relate to pain prevalence? Can variations in these individual- and country-level associations be explained or modified by other contextual factors? In answering these questions, we ultimately assess a more provocative issue of whether it is more advantageous, with respect to pain, to have low wealth and live in a rich country or have high wealth and live in a poor country. We address these questions by combining individual-level data from the World Health Survey with country-level data on wealth and other country-level contextual factors collected from other sources. Our findings help to characterize the global distribution of pain and pain inequalities, and to identify national-level factors that shape pain inequalities.

## 2. Material and methods

### 2.1. Data

Data were obtained from the World Health Survey (WHS), executed by the World Health Organization (WHO) between the years 2002 and 2004 [46,47]. These data were merged with country-level contextual variables [e.g.,^48^]. The WHS was a cross-sectional data collection conducted in 68 countries. The results have been used often for international comparative studies on health [e.g., [49-51]]. The WHS used a nationally-representative multistate cluster sampling design stratified by gender, age, and urban/rural residence, as based on WHO guidelines, in all but a few countries. It administered a standardized translated questionnaire. For most countries, data include sampling weights that account for design and non-response, which we apply as directed [47]. Of the 68 countries, 17 were excluded from this analysis for the following reasons: one did not make data publicly available; nine did not include sampling weights; there were high rates of missing data for five countries; one did not collect information on place of residence (rural vs. urban); one country (Comoros) was found to be an outlier with a country-level pain prevalence of 3.7 standard deviations above the mean. The remaining 51 countries have a total sample size of 233,366 individuals aged 18+. From this total we deleted 2158 with missing weights, 1023 with missing pain information, and 1686 with missing data on other demographic or wealth items used in the analysis, for a total valid sample of 228,499. The total sample sizes by country are provided in Supplementary Materials A.

### 2.2. Measures

Pain: Participants reported experiencing pain in the WHS by answering the following question: “Overall in the last 30 days, how much bodily aches or pains did you have?” with answer categories of “none, mild, moderate, severe, or extreme.” To capture meaningful levels, we dichotomized the response into none or mild versus moderate, severe, or extreme (labeled pain). The ‘no worse than mild pain’ category has been found to be indicative of analgesic control and carry significant health and economic benefits to patients [52]. Sensitivity analyses were conducted to compare cross-country prevalence resulting from alternate measure dichotomizations and no meaningful differences were found.

Individual-level Wealth (ILW): The WHS did not gather direct wealth or consumption information but did ask about amenities that may or may not be present in the household, such as a television, car, computer, refrigerator, etc. We constructed a measure of individual-level wealth (ILW) by using the Filmer Pritchett method for *estimating wealth without expenditure data* [53]. This method provides a prediction of an individual's wealth relative to others using a linear combination of principal component factor scores associated with the presence or absence of household amenities. Specific amenities differed depending on the categorization of the country as higher or lower income. Specific items for each country grouping are shown in Supplementary Materials B. We then took that index and standardized it using the Relative Index of Inequality calculation, or RII [54,55]. RII provides each case a numerical value between 0 and 1 based on its relative position in the distribution of wealth in that country. The measure therefore assumes wealth is an unobservable construct estimated by relative position within a hierarchy. Because wealth, as measured by amenities, has a different meaning for urban versus rural residents, especially in lower income countries, we created separate RII scores for people living in rural and in urban areas of a country.

Country-level Wealth (CLW): To measure country-level wealth (CLW) we took the Gross National Income per capita (GNI) as reported in the year of data collection [48], and calculated the subsequent RII score, between 0 and 1, which is given to each individual observation living in that country. Therefore, like ILW, the CLW measure indicates relative position in the distribution of country-wealth. Using RII for both CLW and ILW makes these two scales similarly standardized and analytically comparable.

Demographic characteristics: Regressions adjusts for age, sex and rural/urban residence. Age is treated as continuous in regressions but is categorized for presentation purposes for some analyses.

Country-level contextual variables: When putting together a set of country-level contextual variables, there are a number of collinearity issues to address. We initially examined a larger set of potential variables and then selected those that were not overly correlated with any other variable. In addition, we selected variables to represent the socioeconomic, political, cultural, and environmental characteristics of countries. We ultimately included eight co-variates, with correlation coefficients between them ranging from a low of about zero to a high of about 0.60, which have been implicated in past research as determinants of population health, and specifically pain prevalence, and represent distinctive constructs. Data come from the World Bank's World Development Indicators [48], and are taken from the year of the WHS or the closest year available. The eight covariates, with variable names and descriptions, are:

1. Gini Index (*gini*): within-country income inequality ranging from 0 to 100.
2. Population density (*density*): number of persons per square kilometer.
3. Average years education (*education*): average years of education for individuals aged 25+.
4. % labor force unemployed (*unemployed*): measured as percent of the total labor force.
5. Poverty ratio (*poverty*): % living below the poverty line defined by World Bank in 2003.
6. Health expenditures as a percent of GDP (*health%GDP*): as reported in U.S. dollars.
7. Total Labor Force Participation (*TLFP*): % aged 15+ participating in the labor force.
8. CO2 emissions (*CO2*): In metric tons per capita.

We also enter dummy variables for the region where the country resides, using six regions defined by the World Health Organization [56].

### 2.3. Analytical strategy

The analysis comprises several steps. First, we show unadjusted percentage reporting pain across study variables. Second, we estimate logistic regression models to show the association between ILW and pain in each country separately. Third, data is pooled and a series of multilevel logistic regressions are estimated to examine associations between ILW, CLW and pain. Quadratic forms of ILW and CLW are tested for nonlinearity and reported if significant. We test interactions between ILW and CLW, and between these two wealth indicators and country-level contextual covariates to determine how the impact of ILW and CLW are moderated. Multilevel models fit equations in two levels. The first estimates fixed effects, capturing the influence of covariates on chances of reporting pain. Fixed effects may be based on individual or country-level contextual data. The second level assesses random effects. One is country, quantifying the degree to which there is cross-country variance in pain. The second is the slope of ILW, quantifying the idiosyncratic effect of ILW unique to each country. We show results for a series of models, each including different fixed effects plus the two random effects. Estimates are presented as log odds, which center on zero such that a positive coefficient indicates that higher wealth associates with higher chances of pain, and a negative coefficient indicates that higher wealth associates with lower chances of pain. Procedures were performed using Stata version 17 [57].

Multilevel runs do not converge when using a full sample of over 228,000 observations, which is common in multilevel models with samples this large [58]. After a number of supplementary analyses to test for model robustness, including running the full sample but with simplified models and running the full sample using other software where models do converge, we determined that a 50% subsample offers consistent and reliable results, and provides more than ample power [59]. The average sample size per country is about 4500, but observations within each varied substantially. To select a subsample, we included all observations from countries with a total sample size under 2690. There are 16 of these. From the remaining 35 countries we randomly selected 2690 observations. This evens out the number of observations per country as best as possible while providing the maximum number from countries with smaller samples. We ran procedures using the selected subsample and the non-selected subsample, and found no meaningful difference, suggesting the reduced analytical sample is a suitable representation of the total. The reduced sample sizes by country are shown in Supplementary Materials A.

To interpret multilevel results, we completed a final step by using results from the model determined to be the best fit and calculating predicted probabilities of pain across specific values of ILW and CLW, holding other covariates constant. The predicted values are calculated using the postestimation *margins* procedure in Stata version 17 [60]. It turns out that the interaction between CLW and Gini is statistically significant, and as such, predicted probabilities are calculated at specific ILW and CLW levels across countries with a low versus high Gini index.

## 3. Results

### 3.1. Unadjusted pain prevalence

Table 1 provides descriptive statistics for study variables for total and reduced sample. For country-level contextual variables, descriptives show means and standard deviations, but in regression models, all country-level contextual variables, except for CLW, are normalized to have a mean of 0 and standard deviation of 1, making it easier to compare magnitudes. All contextual data, by country, including GNI, is provided in Supplementary Materials C. The mean age of the sample is 39, about 51% are female and about 54% live in rural areas.

Table 2 presents unadjusted percent reporting pain across study variables. About 27% report pain. Prevalence is higher for women than men, increases with age, and is only slightly but significantly higher for rural than urban residents. To descriptively show the unadjusted association between ILW, CLW and pain reporting, the sample is divided, as equally as possible, into ILW and CLW quintiles. (Because countries have different numbers of observations, division into quintiles is particularly uneven for CLW.) There is a clear and strong gradient in pain by ILW quintile. Those in the lowest wealth quintile have a prevalence around 33%, and this decreases steadily to about 22% for the wealthiest. For CLW, the relationship is nonlinear; roughly upside-down-U shaped. Countries in the lowest and highest quintiles have the lowest prevalence, while those in the middle quintiles have the highest.

### 3.2. Separate country-specific regressions

**Fig. 1.**
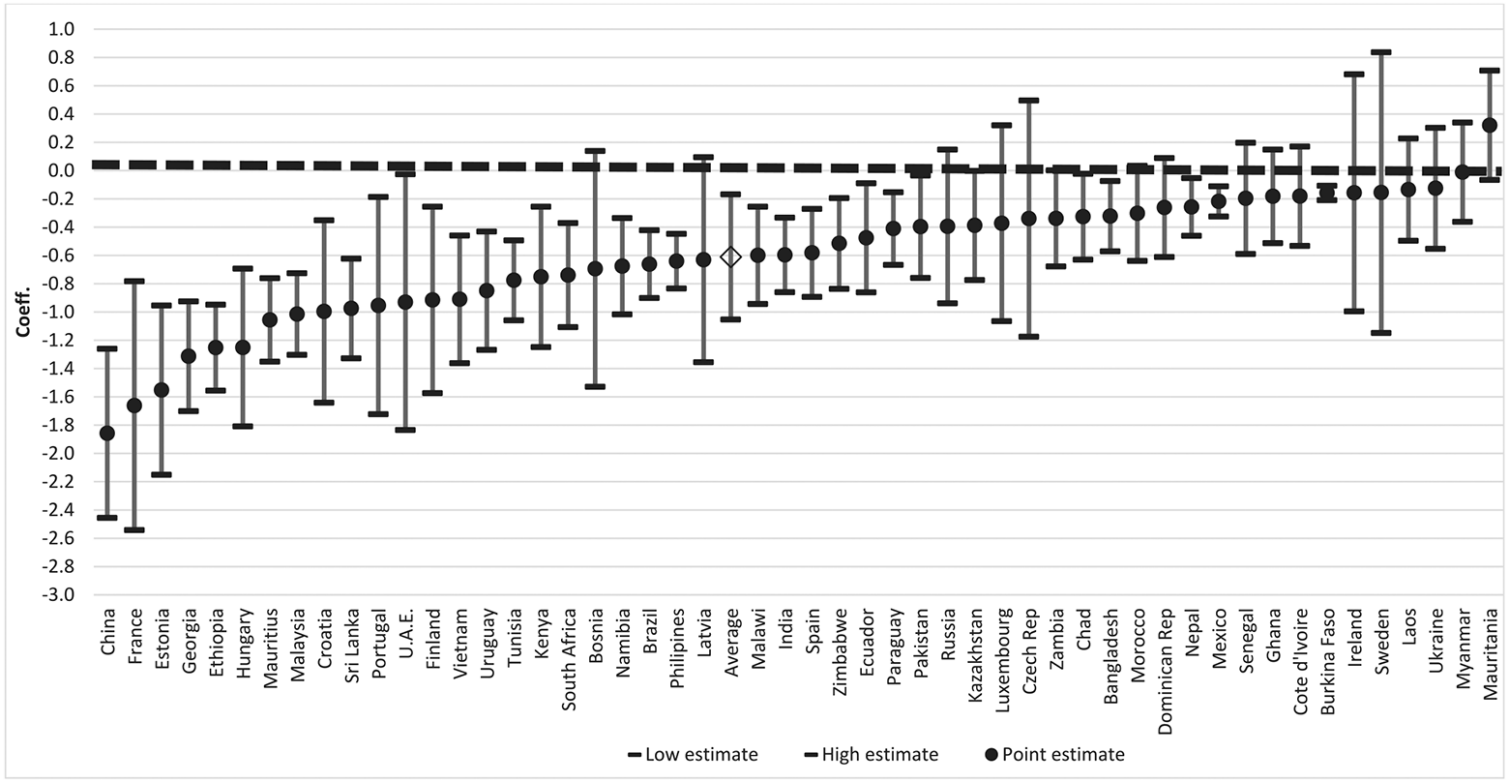
Log-odds of reporting pain by individual-level wealth, from individual regressions separately estimated for 51 countries, showing point estimates and 95% confidence intervals, controlling for age, sex and rural/urban residence^1^. ^1^Estimated from full country samples; arranged from lowest to highest log-odds.

Fig. 1 presents results of separate country-specific regressions of pain on ILW controlling for age, sex and rural/urban residence. (Exact estimates and standard errors are provided in Supplementary Materials D.) There is a clear gradient in most countries. In all by one, the association is negative, meaning higher relative wealth predicts lower chances of reporting pain. In a majority of countries, the negative association is statistically significant. In the one country where the coefficient is positive, the association is non-significant. The average log odds across countries is −0.61 (95% CI −1.05 to −0.17), indicating that on balance there is a strong negative significant association. The association is strongest in China, France, Estonia, Georgia, Ethiopia, Hungary, Mauritius and Malaysia—countries representing several different world regions.

### 3.3. Multilevel models

The multilevel regression results are shown in Table 3. Model 1 includes the individual-level fixed effects plus random effects. There is a strong negative association between ILW and pain worldwide. There is also a substantial and significant random constant, meaning variation exists in the prevalence across countries, and a substantial and significant random slope, meaning there is significant variation in the association between ILW and pain across countries.

CLW is added in Model 2. The association between CLW and pain is nonlinear, as shown by the significant quadratic term. The main effect is strongly positive, while the effect of CLW-squared is strongly negative, indicating that increasing CLW at *lower* levels associates with increasing pain prevalence, while further increases at *higher* levels predict either a diminishing gradient or even a negative association, depending on the magnitude of CLW. This is consistent with the upside-down-U-shaped association found in unadjusted results.

Model 3 adds region and Model 4 adds country-level contextual factors. Net of other covariates, pain is significantly higher in Europe and Africa relative to Western Pacific, which has the lowest prevalence and is the reference. For country-level contextual variables, two are statistically significant: gini and density. In Model 5 just these two country-level contextual variables are included, as only they improve model fit. Higher income inequality, indicated by gini, significantly increases the probability of reporting pain. Higher density does the same.

We next tested interactions between ILW and CLW, and between CLW, ILW and all country-level contextual variables. The only significant interaction is between CLW/CLW-squared and gini. Model 6 includes these. This model is the best fitting of the ones presented. In this model, the association of CLW and pain differs quite a lot by gini. Specifically, a positive gini*CLW coefficient and a negative gini*CLW-squared coefficient suggest that as gini increases so too does the nonlinearity of the CLW-pain association.

### 3.4. Predicted probabilities

**Fig. 2.**
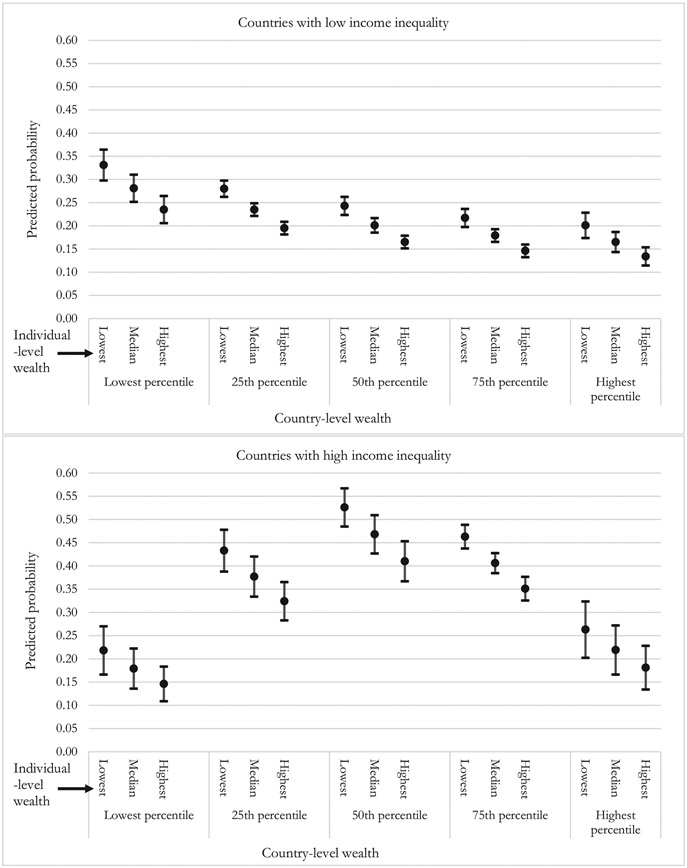
Predicted probability of reporting pain by country-level and individual-level wealth, for countries with low (top panel) and high (bottom panel) income inequality.

Fig. 2 presents predicted probabilities, with 95% confidence intervals, calculated using coefficients from Model 6 in Table 3. The same results are shown in tabular form in Supplementary Materials E. The top panel shows results for countries that have low-income inequality, and the bottom for countries with high income inequality, with these two quantities being set at the same value as the lowest and highest gini scores in the dataset (Sweden and South Africa; unstandardized gini of 25.3 and 64.8, respectively). That is, the two panels show the associations between ILW, CLW and the probability of reporting pain when income inequality is at analytical maximum and minimum values. We set ILW at values of 0.00, 0.50 and 1.00, which means we examine the difference between having the lowest, the median, and the highest wealth within a country and rural/urban region. For CLW, we set values at percentile points 0.00 (lowest possible CLW), 0.25, 0.50, 0.75 and 1.00 (highest possible CLW), which adequately demonstrate the nonlinear nature of the association.

Referring to the top panel, in a country with little income inequality (that is, relatively egalitarian countries) and the *highest percentile* CLW, the predicted probability of pain ranges from about 0.20 for those with lowest ILW, about 0.17 for those with the median ILW, and about 0.13 for those with the highest ILW. For those in a relatively egalitarian country with the *lowest percentile* of CLW, the probabilities range from about 0.33 for those with lowest ILW, 0.28 for those with the median ILW, and 0.24 for those with the highest ILW. Clearly there is a strong within-country wealth gradient in pain reporting, regardless of CLW. Looking at country-level associations in countries with little income inequality, the association of CLW and pain is negative, so that residents of countries with higher wealth are less likely to report pain. Specifically, the predicted probabilities for those with the lowest possible ILW are about 0.33, 0.28, 0.24, 0.22 and 0.20 going from lowest to highest CLW percentile. This is a non-monotonic decline. Overall, in relatively egalitarian countries, pain prevalence is lowest for rich residents of rich countries, and highest for poor residents of poor countries. Moreover, it is a little more favorable to be poor and living in a rich egalitarian country (probability of pain about 0.20) than to be rich in a poor egalitarian country (probability of pain about 0.24). To provide a more concrete idea of which countries fall into which categories of wealth and income-inequality, Supplementary Materials F divides the 51 countries into highest, middle and lowest income-inequality and wealth groupings. Examples of egalitarian wealthy countries are Sweden and Croatia, and egalitarian poor countries are Laos and Ethiopia. Our models then suggest that poor residents in Sweden and Croatia have slightly lower risk of pain than rich residents of Laos and Ethiopia.

Referring to the bottom panel, when within-country income inequality is very high, (that is, non-egalitarian countries) there remains a robust ILW gradient. For instance, in a non-egalitarian country that has the lowest percentile of CLW, the predicted probability of reporting pain is about 0.22 for those with the lowest ILW, about 0.18 for those with the median ILW, and about 0.15 for those with the highest ILW. But the association between CLW and pain is highly nonlinear in these non-egalitarian countries, such that the highest probability of pain exists for people in middle level CLW countries. To provide an example of the nonlinearity, the probabilities of pain for those with median ILW are about 0.18, 0.38, 0.47, 0.41 and 0.22, going from lowest to highest CLW percentile. In these countries, it is better to be wealthy and live in a country with low CLW (probability of pain about 0.15) than to be poor and in a country with high CLW (probability of pain about 0.26). Looking at Supplementary Materials F, examples of non-egalitarian rich countries are Mexico and Malaysia while non-egalitarian poor countries include Ghana and Nepal. Our models then suggest that rich residents Ghana and Nepal are better off than poor residents in Mexico and Malaysia. However, the highest probability of pain is found amongst poor people living in non-egalitarian middle-income countries, examples of which, as seen in Supplementary Materials F, are South Africa and Brazil.

## 4. Discussion

In an effort to understand global socioeconomic gradients in pain, this study posed three research questions and addressed them using 51-country data from the World Health Survey. The first two questions asked whether individual-level wealth (ILW) and country-level wealth (CLW) associate with the probability of reporting pain. We found that in all parts of in the world, across regions and levels of development, higher ILW is protective, although the strength of the association varies. In country-specific regressions, more wealth associated with lower probabilities of pain in each country except one. A negative association was statistically significant at *p* < .05 in 34 countries. Individual-level socioeconomic disparities in pain have been found by others in more limited contexts [e.g., [61,62-65]]. Literature that compares this association cross-nationally is much rarer, although Todd et al. [66] came to similar conclusions for selected countries in Europe. For instance, they showed that prevalence of back/neck pain in some European countries can vary by more than 10 percentage points comparing those with high versus low education. Our findings show that an association between individual-level wealth and pain extends to all world regions, such as across Asia and Africa.

The association between CLW and pain, in contrast, was found to be nonlinear and moderated by within-country income inequality as measured by the Gini index. In more egalitarian countries, there is a decline in the probability of reporting pain by CLW such that living in a richer country is protective. But, in countries with high income inequality, the association is nonlinear such that people in middle-income countries have the highest probability of reporting pain. This is a somewhat surprising finding but not without precedent. Both obesity and diabetes, for instance, have been found to be higher in middle-income as opposed to lower-income countries [67,68]. Both obesity and diabetes have been strongly linked to pain prevalence [69,70]. Moreover, a study by Navarro and Shi [71] found countries with political traditions committed to redistribution of wealth tend to be more successful in improving health conditions. The argument in these examples is that the middle stages of economic growth can bring with it new behaviors and lifestyles detrimental to health, while public and formal health care structures are still underdeveloped, and there is little political impetus for health spending.

The third asked whether variations could be explained or modified by contextual factors. We found that eight such factors plus region did little to reduce the association of ILW and pain or the random effects. We did find two country-level contextual factors had independent influences. First, pain is generally higher in countries with greater income inequality. For instance, Model 5 in Table 3 indicates a log-odds for the Gini index of 0.213 (*p* < .01). While there is debate in the literature regarding the impact of income inequality on health [31,33], the finding is here consistent with the side of the argument that believes populations benefit from more egalitarian income distributions. There are a number of explanations for this. Egalitarian societies may provide a larger pool of people with the means to access health resources, or with opportunities to access other resources that benefit health. Also, high levels of inequality may contribute to psychosocial stress levels, which are likely to impact pain outcomes [72]. Second, we also found reported pain to be higher in countries with high population density. The negative health effects of high population density have been expounded upon by the World Health Organization [73], which suggests that crowded urban environments lead to inadequate housing, hygiene problems, high pollution levels, lack of space for active living, and greater perceived stress, all of which associate with many physical health conditions.

We also provocatively asked whether, when it comes to risk of pain, it is better to be poor and living in a rich country or rich and living in a poor country. It turns out that the answer depends on national income inequality. In countries that are more egalitarian, the probability of pain is lowest among rich people in rich countries and highest among poor people in poor countries. In these egalitarian countries, it is somewhat better to be poor and live in a rich country than the opposite. In contrast, when income inequality is high, the pain-CLW association is nonlinear (upside-down-U shaped). It is better to be rich in a poor country than the opposite, but those at greatest risk of reporting pain are the poor living in non-egalitarian middle-income countries.

Although there is little if any previous research that has examined the issue of wealth and pain on a country-level across these many countries, there are two earlier studies that provide particularly relevant context for our findings. In a strictly macro-level analysis, Biggs et al. [34] studied the connection between country-level wealth and population health measures, like life expectancy, over time across 22 Latin American countries. They found that while higher wealth, measured as GDP, significantly benefitted population health outcomes, the association waned at higher levels of income inequality. Our findings are generally in concert with these given that Latin American countries tend to be on the higher end of income inequality. Semynov et al. [35] assessed whether individual- and country-level economic resources associate with a single broad indicator of individual health across 14 European countries plus Israel and the U.S. These authors found little moderating impact of income inequality except for in the U.S. Our study is generally in agreement here as well since we found that the association is fairly consistent across low-income inequality countries, which would include the most of the countries of Europe.

There are several limitations to our study. The WHS is an older dataset, which limits current generalizability. It is, however, the only dataset that permits examination of the determinants of pain across such a large and diverse set of countries. The WHS included only a single question on pain and does not allow an opportunity to examine more detailed aspects such as chronic versus acute pain. It is unfortunate that a number of countries available in the WHS were deemed unusable because of missing weight information or missing data on key variables. Finally, the cross-sectional nature of the dataset makes it difficult for definitive statements regarding causality. These limitations provide impetus for future studies. In particular, establishing associations using more contemporary data and between other socioeconomic characteristics and pain would help confirm the generalizability of these findings. Further, it is necessary to consider broader definitions of pain, particularly using measures that affirm its chronic nature.

Pain is a global health problem that has a profound impact on quality of life [19,74-77]. It is gaining attention as an international public health concern [2,5]. There have indeed been calls to consider access to pain treatment a human right [78]. The ubiquitous nature of pain, its implications for policy and its link to other conditions including disability and mortality make pain a compelling measure of population health [3]. In this context, we believe our findings shed light on the nature and extent of inequalities in pain, and contribute to broader scholarship on socioeconomic factors as social determinants of health. Further, there is growing evidence suggesting that pain risk is shaped by economic distress and other forms of despair [79,80]. These arguments mostly focus on the U.S., but there are international examples linking economic distress, poverty, food insecurity, and other factors that correlate with low social position to risk of physical pain [e.g., [61,63,81-83]]. Our study adds to this literature by showing the nearly-universal link between personal (within-country) wealth and pain.

## 5. Conclusion

Inequalities in pain by individual-level wealth are observed in countries worldwide. Countries with higher country-level wealth have lower pain prevalence. But the exact nature of the association between country-level wealth and pain depends on the moderating influence of country-level income inequality, measured by the Gini index. Pain prevalence is highest in non-egalitarian countries with middle-level national wealth.

## Supplementary Material

Appendix A. Supplementary data

## Figures and Tables

**Table 1:** Variable descriptives, showing weighted means and standard deviations or percentages.

		Total sample (*N* = 228,499)		Reduced sample^1^ (*N* = 114,289)	
	Abbr.	Mean or %	Std. Dev	Mean or %	Std. Dev
Individual-level measures					
Individual-level wealth	ILW	0.50	0.29	0.50	0.29
Age		39.0	16.3	39.7	16.6
Rural		54.1%		51.1%	
Female		50.7%		51.1%	
Country-level measures					
Country-level wealth	CLW	0.50	0.29	0.52	0.30
World region					
Western Pacific	WPac	10.1%		9.4%	
Southeast Asia	SEA	26.2%		18.8%	
Europe	Eur	16.2%		21.4%	
Africa	Afr	17.5%		13.0%	
Americas	Amer	14.2%		12.69%	
East Mediterranean	EMed	15.8%		24.5%	
Contextual variables					
Gini index	Gini	40.0	9.5	39.7	10.3
Persons per square kilometer	density	225.1	287.1	197.3	278.5
Average years education age 25+	education	6.0	2.6	6.5	2.9
of labor force unemployed	unemployed	6.1	5.6	7.3	6.9
Poverty gap ratio	poverty	8.3	6.1	7.6	6.5
Health expenditures as a percent of GDP	health% GDP	4.9	2.2	5.3	2.4
Total labor force participation	TLFP	61.6	8.9	61.7	9.3
CO^2^ emissions	CO2	2.9	5.3	3.9	6.8

1 This is a 50% sample chosen randomly for analytic purposes. See Appendix A for further details.

**Table 2:** Pain prevalence by several key study variables and 95% confidence intervals, full sample^a^.

Variable	Categories	Unweighted N	Pain % (95% CI)
Total sample		228,498	26.75 (26.57–26.94)
Gender	Women	126,995	32.46 (32.20–32.72)
	Men	101,504	20.88 (20.63–21.13)
Age (in categories)	18–29	66,785	16.70 (16.42–16.98)
	30–39	55,311	23.28 (22.92–23.63)
	40–49	41,802	27.86 (27.43–28.29)
	50–59	27,600	35.22 (34.66–35.78)
	60–69	20,029	45.02 (44.34–45.71)
	70+	16,972	55.04 (54.29–55.79)
Rural/urban residence	Rural	112,917	27.39 (27.13–27.65)
	Urban	115,582	26.00 (25.75–26.26)
Individual-level wealth (ILW)	Lowest Quintile	45,719	33.25 (32.82–33.68)
	Quintile 2	45,749	29.00 (28.58–29.41)
	Quintile 3	45,632	26.78 (26.38–27.19)
	Quintile 4	45,725	24.98 (24.58–25.38)
	Highest Quintile	45,674	21.98 (21.60–22.36)
Country-level wealth (CLW)	Lowest Quintile	47,153	19.61 (19.25–19.69)
	Quintile 2	48,025	27.01 (26.61–27.41)
	Quintile 3	42,665	32.41 (31.97–32.86)
	Quintile 4	76,637	29.06 (28.74–29.38)
	Highest Quintile	14,019	23.20 (22.50–23.90)

a Pain prevalence is sample-weight adjusted. Pain defined as answering ‘moderate, severe or extreme’ to the question: “Overall in the last 30 days, how much bodily aches or pains did you have?”

**Table 3:** Results for multilevel logit models predicting pain, including within- and between-country wealth indexes (N = 114,289)^1^.

	Model 1	Model 2	Model 3	Model 4	Model 5	Model 6
Fixed effects						
Individual-level						
ILW	−0.545**	−0.545**	−0.577**	−0.582**	−0.546**	−0.546**
Gender	0.583**	0.583**	0.583**	0.583**	0.583**	0.583**
Age	0.037**	0.037**	0.037**	0.037**	0.037**	0.037**
Rural	0.207**	0.207**	0.207**	0.206**	0.206**	0.206**
Country-level						
CLW		2.097*	2.384**	2.026	1.204	2.004*
CLW-squared		−2.065*	−2.180**	−1.884†	−1.465*	−2.322**
World regions (vs. Western Pacific)						
Southeast Asia			0.551	0.269	0.143	0.170
Europe			0.522	1.034**	0.968**	1.053**
Africa			0.753**	0.746*	0.674*	0.681*
Americas			0.608	0.461	0.561†	0.566†
Eastern Mediterranean						
Contextual factors^2^			0.263	0.515	0.804†	0.870*
gini				0.252*	0.213**	−0.138
density				0.254**	0.261**	0.257**
education				−0.083		
unemployed				0.010		
poverty				−0.061		
health%GDP				0.012		
TLFP				0.102		
CO2				0.000		
Interactions						
Gini*CLW						1.518*
Gini*CLW^2^						−1.297*
Constant	−2.910	−3.228	−3.884	−3.835	−3.517	−3.696
Random effects						
σ Constant	0.244**	0.215**	0.193**	0.155**	0.142**	0.133**
σ Slope	0.125**	0.125**	0.114**	0.115**	0.126**	0.127**
LPL	−58,537.5	−58,534.7	−58,526.9	−58,521.9	−58,524.9	−58,523.6

** p < .01

* 0.01 < p < .05

† 0.05 < *p* < .10.

1 This analysis uses the reduced sample.

2 Contextual variables are normalized to have a mean of 0 and standard deviation of 1.
