# Supplementary material for: A global comparative study of wealth-pain gradients: Investigating individual- and country-level associations
Source: Dialogues Health. Author manuscript; Available in PMC 2024 Dec 1. (PMC10718570; doi:10.1016/j.dialog.2023.100122)
Supplement: Appendix A. Supplementary data [file NIHMS1946042-supplement-Appendix_A__Supplementary_data.docx]

Supplementary Materials A. Respondents in each country in the analysis, organized by region^1^

| **Region/Country** | **Total N** | **50% subsample N**^1^ |
| --- | --- | --- |
| **Europe** |  |  |
| Bosnia | 1,028 | 1,028 |
| Croatia | 988 | 988 |
| Czech Republic | 915 | 915 |
| Estonia | 1,006 | 1,006 |
| Finland | 1,013 | 1,013 |
| France | 993 | 993 |
| Georgia | 2,692 | 2,690 |
| Hungary | 1,398 | 1,398 |
| Ireland | 977 | 977 |
| Kazakhstan | 4,493 | 2,690 |
| Latvia | 852 | 852 |
| Luxembourg | 700 | 700 |
| Portugal | 1,024 | 1,024 |
| Russia | 4,387 | 2,690 |
| Spain | 6,232 | 2,690 |
| Sweden | 994 | 994 |
| Ukraine | 2,395 | 2,395 |
|  |  |  |
| **Americas** |  |  |
| Brazil | 4,828 | 2,690 |
| Dominican Rep. | 4,522 | 2,690 |
| Ecuador | 4,439 | 2,690 |
| Mexico | 38,506 | 2,690 |
| Paraguay | 5,126 | 2,690 |
| Uruguay | 2,780 | 2,690 |
|  |  |  |
| **Eastern Mediterranean** |  |  |
| Morocco | 4,463 | 2,690 |
| Pakistan | 6,078 | 2,690 |
| Tunisia | 5,033 | 2,690 |
| United Arab Emirates | 1,171 | 1,171 |
|  |  |  |
| **Africa** |  |  |
| Burkina Faso | 4,800 | 2,690 |
| Chad | 4,561 | 2,690 |
| Côte d'Ivoire | 3,101 | 2,690 |
| Ethiopia | 4,917 | 2,690 |
| Ghana | 3,893 | 2,690 |
| Kenya | 4,330 | 2,690 |
| Malawi | 5,203 | 2,690 |

Continued on next page

Supplementary Materials A: Continued

| **Region/Country** | **Total N** | **50% subsample N**^1^ |
| --- | --- | --- |
| Mauritania | 3,596 | 2,690 |
| Mauritius | 3,877 | 2,690 |
| Namibia | 3,975 | 2,690 |
| Senegal | 2,420 | 2,420 |
| South Africa | 2,265 | 2,265 |
| Zambia | 3,787 | 2,690 |
| Zimbabwe | 3,982 | 2,690 |
|  |  |  |
| **South-East Asia** |  |  |
| Bangladesh | 5,541 | 2,690 |
| India | 9,560 | 2,690 |
| Myanmar | 5,886 | 2,690 |
| Nepal | 8,683 | 2,690 |
| Sri Lanka | 6,495 | 2,690 |
|  |  |  |
| **Western Pacific** |  |  |
| China | 3,993 | 2,690 |
| Lao PDR | 4,880 | 2,690 |
| Malaysia | 6,005 | 2,690 |
| Philippines | 10,061 | 2,690 |
| Vietnam | 3,465 | 2,690 |
|  |  |  |
| **Total** | 228,499 | 114,289 |

^1^ The 50% subsample is constructed to facilitate convergence of multilevel models. It is put together as follows: From countries with 2,690 or fewer observations, all observations are included in the subsample. From countries with more than 2,690 observations, 2,690 observations are randomly selected.

Supplementary Materials B. Individual-level wealth items in low- and high- income countries^1^

| **World Health Survey Permanent Income Indicators** | |
| --- | --- |
| **Low Income Country Wealth Index Items** | **High Income Country Wealth Index Items** |
|  |  |
| Can you please tell me how many rooms there are in your home?* | Can you please tell me how many rooms there are in your home?* |
| How many chairs are there in your home? | How many cars are there in your household?* |
| 1. How many tables are there in your home? | 1. How many televisions are there in your household? |
| 1. How many cars are there in your household?* | 1. Does anyone in your home have a bicycle?* |
| 1. Does your home have electricity? | 1. Does anyone in your home have a VCR? |
| 1. Does anyone in your home have a bicycle?* | 1. Does anyone have a stereo system? |
| 1. Does anyone have a clock? | 1. Does anyone have a DVD player? |
| 1. Does anyone have a bucket? | 1. Does anyone have a video camera? |
| 1. Does anyone have a washing machine for clothes?* | 1. Does anyone have a washing machine for clothes?* |
| 1. Does anyone have a washing machine for dishes?* | 1. Does anyone have a washing machine for dishes?* |
| 1. Does anyone have a refrigerator?* | 1. Does anyone have a vacuum cleaner? |
| 1. Does anyone have a fixed line telephone?* | 1. Does anyone have a refrigerator?* |
| 1. Does anyone have a mobile/ cellular telephone?* | 1. Does anyone have a fixed line telephone?* |
| 1. Does anyone have a television? | 1. Does anyone have a mobile/ cellular telephone?* |
| 1. Does anyone have a computer?* | 1. Does anyone have a computer?* |
|  | 1. Does anyone have access to the internet/ World Wide Web from your home? |
|  | 1. Does anyone have any subscriptions to magazines and/or newspapers? |
|  | 1. Does anyone have a security system in your home (alarm, reinforced doors, guards, etc.)? |
|  | 1. Do you employ anybody in your house who is not a member of your family (gardener, cook, cleaning lady, driver, etc.)? |
|  | 1. Do you have a second home? |

^1^Survey items that are the same between both low- and high-income household surveys are indicated by an asterisk.

Supplementary Materials C: Country-level contextual data by country

| Country | Gross National Income | Gini | density | education | unemployed | poverty | health%GDP | TLFP | CO2 |
| --- | --- | --- | --- | --- | --- | --- | --- | --- | --- |
| Bangladesh | 460 | 33.2 | 1035.5 | 4.4 | 4.3 | 5.0 | 2.2 | 57.6 | 0.26 |
| Bosnia | 2040 | 34.0 | 73.5 | 7.2 | 28.7 | 0.2 | 8.0 | 44.4 | 3.85 |
| Brazil | 2980 | 57.6 | 21.8 | 6.2 | 10.0 | 9.5 | 8.2 | 65.0 | 1.77 |
| Burkina Faso | 320 | 43.3 | 46.3 | 1.3 | 2.8 | 23.8 | 3.5 | 75.6 | 0.09 |
| Chad | 210 | 39.8 | 7.4 | 1.4 | 1.2 | 26.6 | 5.5 | 72.3 | 0.08 |
| China | 1280 | 42.0 | 137.2 | 6.7 | 4.6 | 24.6 | 4.4 | 75.0 | 3.52 |
| Cote d'Ivoire | 620 | 41.3 | 55.3 | 9.8 | 5.2 | 7.7 | 4.5 | 63.9 | 0.31 |
| Croatia | 6670 | 32.6 | 77.0 | 11.4 | 13.9 | 1.7 | 6.4 | 52.3 | 5.34 |
| Czech Republic | 8110 | 26.6 | 131.9 | 3.5 | 7.5 | 0.1 | 6.6 | 59.4 | 11.97 |
| Dominican Republic | 2640 | 52.1 | 183.2 | 6.8 | 6.9 | 6.1 | 4.4 | 57.8 | 2.43 |
| Ecuador | 2110 | 53.4 | 53.8 | 7.1 | 5.7 | 12.5 | 5.4 | 66.4 | 2.02 |
| Estonia | 5730 | 37.2 | 32.3 | 11.9 | 11.3 | 4.7 | 4.9 | 58.8 | 12.49 |
| Ethiopia | 110 | 29.8 | 72.2 | 1.7 | 2.9 | 9.4 | 4.9 | 81.4 | 0.07 |
| Finland | 28720 | 27.7 | 17.1 | 9.6 | 10.5 | 3.1 | 7.7 | 61.0 | 13.17 |
| France | 25930 | 31.4 | 113.6 | 10.2 | 8.3 | 0.2 | 10.1 | 55.9 | 6.12 |
| Georgia | 990 | 36.7 | 69.1 | 12.0 | 11.5 | 10.0 | 8.3 | 65.4 | 0.96 |
| Ghana | 320 | 42.8 | 91.2 | 6.3 | 7.4 | 8.0 | 2.8 | 73.0 | 0.37 |
| Hungary | 6630 | 29.9 | 113.0 | 10.5 | 5.8 | 0.6 | 8.1 | 49.6 | 5.83 |
| India | 520 | 34.4 | 373.8 | 4.7 | 5.7 | 9.8 | 4.0 | 57.9 | 0.99 |
| Ireland | 29890 | 32.9 | 58.0 | 11.4 | 4.5 | 0.2 | 7.0 | 62.8 | 10.68 |
| Kazakhstan | 1810 | 33.7 | 5.5 | 11.2 | 8.8 | 4.9 | 3.7 | 69.8 | 9.54 |
| Kenya | 400 | 46.5 | 60.9 | 5.6 | 2.9 | 16.4 | 5.2 | 67.6 | 0.20 |
| Laos | 340 | 32.6 | 24.2 | 4.1 | 1.6 | 7.4 | 4.5 | 79.4 | 0.22 |
| Latvia | 4670 | 36.4 | 36.8 | 11.2 | 12.1 | 5.4 | 5.5 | 57.0 | 3.22 |
| Luxembourg | 46000 | 30.2 | 185.9 | 10.9 | 3.7 | 0.1 | 7.0 | 53.2 | 21.93 |

Continued on next page

Supplementary Materials C: Continued

| Country | Gross National Income | Gini | density | education | unemployed | poverty | health%GDP | TLFP | CO2 |
| --- | --- | --- | --- | --- | --- | --- | --- | --- | --- |
| Malawi | 220 | 39.9 | 127.3 | 3.3 | 6.0 | 31.8 | 4.7 | 77.6 | 0.08 |
| Malaysia | 4160 | 46.1 | 75.2 | 8.0 | 3.6 | 3.2 | 3.0 | 61.3 | 6.41 |
| Mauritania | 710 | 40.2 | 2.8 | 3.2 | 9.9 | 3.6 | 5.3 | 49.0 | 0.49 |
| Mauritius | 4410 | 35.7 | 597.7 | 6.8 | 8.3 | 5.1 | 3.2 | 58.6 | 2.52 |
| Mexico | 7360 | 50.1 | 53.0 | 7.1 | 3.5 | 16.2 | 5.8 | 59.2 | 4.27 |
| Morocco | 1540 | 40.6 | 66.7 | 3.7 | 11.9 | 7.1 | 4.6 | 50.3 | 1.26 |
| Myanmar | 180 | 38.1 | 73.7 | 3.4 | 0.8 | 1.1 | 2.0 | 70.2 | 0.20 |
| Namibia | 2290 | 63.3 | 2.3 | 5.8 | 21.7 | 25.1 | 10.0 | 55.6 | 1.00 |
| Nepal | 260 | 43.8 | 175.0 | 2.6 | 1.7 | 16.5 | 4.4 | 85.1 | 0.11 |
| Pakistan | 570 | 32.5 | 198.6 | 4.0 | 0.6 | 3.5 | 2.4 | 51.3 | 0.78 |
| Paraguay | 1230 | 54.9 | 14.2 | 7.0 | 6.8 | 6.9 | 4.3 | 68.6 | 0.72 |
| Philippines | 1170 | 46.6 | 278.5 | 8.0 | 3.5 | 11.9 | 3.2 | 61.6 | 0.86 |
| Portugal | 13260 | 38.7 | 114.3 | 7.2 | 6.1 | 1.4 | 8.9 | 62.0 | 5.85 |
| Russia | 2590 | 40.0 | 8.8 | 11.4 | 8.2 | 1.6 | 5.2 | 60.2 | 11.09 |
| Senegal | 710 | 41.2 | 54.8 | 2.4 | 6.7 | 16.4 | 4.3 | 51.1 | 0.47 |
| South Africa | 2920 | 64.8 | 38.5 | 8.8 | 32.5 | 20.4 | 6.9 | 55.1 | 7.61 |
| Spain | 18030 | 31.8 | 84.5 | 8.6 | 11.3 | 1.1 | 7.5 | 54.9 | 0.58 |
| Sri Lanka | 940 | 41.0 | 306.6 | 10.2 | 8.2 | 10.2 | 3.8 | 56.6 | 6.12 |
| Sweden | 32750 | 25.3 | 21.8 | 11.8 | 5.6 | 0.5 | 8.5 | 62.5 | 2.14 |
| Tunisia | 2460 | 37.7 | 64.0 | 5.4 | 14.5 | 4.0 | 5.5 | 45.9 | 3.31 |
| Ukraine | 980 | 28.7 | 82.5 | 11.0 | 9.1 | 1.1 | 6.5 | 55.5 | 28.77 |
| United Arab Emerites | 32780 | 32.5 | 52.3 | 8.7 | 3.0 | 0.0 | 2.6 | 76.0 | 1.38 |
| Uruguay | 4250 | 45.0 | 19.0 | 8.0 | 16.7 | 5.1 | 10.4 | 62.2 | 0.96 |
| Vietnam | 500 | 37.0 | 265.4 | 6.0 | 2.3 | 9.8 | 4.7 | 76.3 | 0.00 |
| Zambia | 410 | 42.1 | 15.1 | 6.1 | 14.8 | 18.8 | 7.2 | 79.5 | 0.19 |
| Zimbabwe | 440 | 43.2 | 31.0 | 6.8 | 4.7 | 5.2 | 10.5 | 81.7 | 0.89 |

Supplementary Materials D: Log-odds of reporting pain by individual-level wealth, from individual regressions separately estimated for 51 countries, showing point estimates and standard errors, controlling for age, sex and rural/urban residence

| Country | Coeff. | SE | Country | Coeff. | SE |
| --- | --- | --- | --- | --- | --- |
| China | -1.858** | 0.305 | Czech Republic | -0.340 | 0.426 |
| France | -1.662** | 0.449 | Zambia | -0.339* | 0.173 |
| Estonia | -1.552** | 0.305 | Chad | -0.326* | 0.155 |
| Georgia | -1.313** | 0.198 | Bangladesh | -0.323** | 0.127 |
| Ethiopia | -1.252** | 0.155 | Morocco | -0.302† | 0.172 |
| Hungary | -1.251** | 0.285 | Dominican Republic | -0.262 | 0.179 |
| Mauritius | -1.056** | 0.151 | Nepal | -0.257** | 0.104 |
| Malaysia | -1.015** | 0.147 | Mexico | -0.218** | 0.055 |
| Croatia | -0.996** | 0.329 | Senegal | -0.197 | 0.201 |
| Sri Lanka | -0.975** | 0.180 | Ghana | -0.182 | 0.169 |
| Portugal | -0.955** | 0.392 | Cote d'Ivoire | -0.181 | 0.179 |
| U.A.E. | -0.930** | 0.461 | Burkina Faso | -0.158 | -0.026 |
| Finland | -0.915** | 0.336 | Ireland | -0.158 | 0.428 |
| Vietnam | -0.910** | 0.230 | Sweden | -0.156 | 0.506 |
| Uruguay | -0.850** | 0.214 | Laos | -0.135 | 0.185 |
| Tunisia | -0.776** | 0.144 | Ukraine | -0.125 | 0.218 |
| Kenya | -0.752** | 0.253 | Myanmar | -0.012 | 0.179 |
| South Africa | -0.739** | 0.188 | Mauritania | 0.320 | 0.197 |
| Bosnia | -0.695† | 0.425 |  |  |  |
| Namibia | -0.676** | 0.174 |  |  |  |
| Brazil | -0.662** | 0.122 |  |  |  |
| Philippines | -0.641** | 0.098 |  |  |  |
| Latvia | -0.630† | 0.370 |  |  |  |
| Malawi | -0.599** | 0.176 |  |  |  |
| India | -0.597** | 0.135 |  |  |  |
| Spain | -0.582** | 0.159 |  |  |  |
| Zimbabwe | -0.516** | 0.164 |  |  |  |
| Ecuador | -0.476* | 0.197 |  |  |  |
| Paraguay | -0.410** | 0.131 |  |  |  |
| Pakistan | -0.397* | 0.185 |  |  |  |
| Russia | -0.395 | 0.277 |  |  |  |
| Kazakhstan | -0.388* | 0.197 |  |  |  |
| Luxembourg | -0.373 | 0.353 |  |  |  |

** p < .01 * .01 < p < .05 † .05 < p < .10

Supplementary Materials E: Predicted probability of reporting pain by country- and individual-level wealth, for countries with low- and high-income inequality, showing point estimates and high and low estimates based on 95% confidence intervals^1^

| Income inequality | Country-level wealth | Individual-level wealth | Point estimate | High estimate | Low estimate |
| --- | --- | --- | --- | --- | --- |
| Low | Lowest percentile | Lowest | .331 | .364 | .298 |
|  |  | Median | .281 | .310 | .252 |
|  |  | Highest | .235 | .2164 | .206 |
|  | 25^th^ percentile | Lowest | .280 | .298 | .262 |
|  |  | Median | .235 | .249 | .221 |
|  |  | Highest | .195 | .209 | .181 |
|  | 50^th^ percentile | Lowest | .243 | .263 | .223 |
|  |  | Median | .201 | .217 | .185 |
|  |  | Highest | .165 | .179 | .151 |
|  | 75^th^ percentile | Lowest | .217 | .237 | .197 |
|  |  | Median | .179 | .193 | .165 |
|  |  | Highest | .146 | .160 | .132 |
|  | Highest percentile | Lowest | .201 | .228 | .174 |
|  |  | Median | .165 | .187 | .143 |
|  |  | Highest | .134 | .154 | .114 |
| High | Lowest percentile | Lowest | .218 | .270 | .166 |
|  |  | Median | .179 | .222 | .136 |
|  |  | Highest | .146 | .183 | .109 |
|  | 25^th^ percentile | Lowest | .433 | .478 | .388 |
|  |  | Median | .377 | .420 | .334 |
|  |  | Highest | .324 | .365 | .283 |
|  | 50^th^ percentile | Lowest | .526 | .567 | .485 |
|  |  | Median | .468 | .509 | .427 |
|  |  | Highest | .410 | .453 | .367 |
|  | 75^th^ percentile | Lowest | .463 | .488 | .438 |
|  |  | Median | .406 | .428 | .384 |
|  |  | Highest | .351 | .376 | .326 |
|  | Highest percentile | Lowest | .263 | .323 | .202 |
|  |  | Median | .2189 | .272 | .166 |
|  |  | Highest | .181 | .228 | .134 |

^1^Low and high-income equality determined by Gini. Low is set to the lowest in the dataset, represented by the country Sweden, and high is set to the highest in the dataset, represented by South Africa

Supplementary Materials F: Countries in the analysis divided into tertiles of wealth, as measured by GNI per capita, by income inequality, as measured by the Gini Index^1^

|  | Wealth | | |
| --- | --- | --- | --- |
| Income-inequality | Lowest 1/3 | Middle 1/3 | Highest 1/3 |
| Lowest 1/3 | Bangladesh  Ethiopia  Laos  Pakistan | Ukraine  Kazakhstan  Bosnia | Armenia  Croatia  Czech Republic  France  Finland  Hungary  Ireland  Luxembourg  Spain  Sweden |
| Middle 1/3 | Chad  Cote d’Ivoire  India  Malawi  Mauritania  Myanmar  Vietnam | Georgia  Morocco  Russia  Senegal  Sri Lanka  Tunisia | Estonia  Latvia  Mauritius  Portugal |
| Highest 1/3 | Burkina Faso  Ghana  Kenya  Nepal  Zambia  Zimbabwe | Brazil  China  Dominican Republic  Ecuador  Namibia  Paraguay  Philippines  South Africa | Malaysia  Mexico  Uruguay |

^1^See Supplementary Materials C for specific values of GNI and Gini
